# Korean Medical Consultation With Open-Weight Large Language Models: Pilot Comparative Evaluation of Retrieval-Augmented Generation With Metadata Filtering

**DOI:** 10.2196/72604

**Published:** 2026-04-30

**Authors:** Saeyoun Choi, Donghyun Kim, Ji-Hwan Jeon, Minji Kim, Dong Hun Lee, DaeHwan Ahn, Eu Sun Lee, Yoon Ji Kim, Hyun Youk

**Affiliations:** 1 MAIN Corp, 1 Gangwon-daehak-gil, Room 1201 (Bodeum-gwan), Chuncheon City, Gangwon Province, 24341, Republic of Korea, 82 10-9840-2120; 2 Department of Industrial and Systems Engineering, Dongguk University, Seoul, Republic of Korea; 3 InVisionLab Inc, Seoul, Republic of Korea; 4 Department of Healthcare Management, Gachon University, Seongnam, Republic of Korea; 5 KOI Healthcare Co, Ltd, Seongnam, Republic of Korea; 6 Department of Preventive Medicine, College of Medicine, University of Ulsan, Seoul, Republic of Korea; 7 Digital Health Laboratory, Wonju College of Medicine, Yonsei University, Wonju, Republic of Korea; 8 Regional Trauma Center, Wonju Severance Christian Hospital, Wonju, Republic of Korea

**Keywords:** large language model, LLM, retrieval-augmented generation, RAG, metadata filtering, health chatbot, Korean health care

## Abstract

**Background:**

This study develops an open-source large language model–based chatbot tailored for Korean health consultations. The chatbot was implemented using the retrieval-augmented generation (RAG) technique alongside metadata filtering to enhance its performance.

**Objective:**

This study aims to analyze and compare the performance of a RAG-based chatbot with other leading language models in the context of Korean health consultations.

**Methods:**

A 10.4 GB Korean medical document corpus (487,277 segments) was constructed from official websites of major Korean hospitals, public health sources, and medical textbooks. This study quantitatively compared 5 open-source large language models (Qwen3:4B, Mistral:7B, Llama-3.1:8B, Gpt-Oss:20B, and Gemma3:27B) in 3 configurations: baseline (model only), RAG-only, and RAG with metadata filtering. The RAG system used a specialized Korean embedding model (upskyy/bge-m3-korean) and an Elasticsearch store. Performance was assessed by an emergency medicine specialist using a validation set of 226 questions across 7 common diseases and scoring responses based on accuracy, safety, and helpfulness.

**Results:**

The application of RAG alone failed to yield statistically significant performance improvements and, in some cases (Llama 3.1: 8B and Gemma 3: 27B), resulted in decreased scores. However, the combination of RAG with metadata filtering yielded statistically significant (*P*<.05) performance increases in most models. Notably, the average score for Mistral:7B increased from 3.79, SD 0.08, to 4.10, SD 0.10, and Gpt-Oss:20B increased from 4.43, SD 0.05, to 4.51, SD 0.04, with the latter achieving the highest safety score (4.61, SD 0.03). The Gemma3:27B model, which possessed a high baseline performance (4.42, SD 0.03), was an exception, exhibiting no significant improvement (*P*=.14) even with filtering.

**Conclusions:**

The effectiveness of RAG for specialized domains such as Korean medical consultation is highly dependent on a metadata filtering process that controls the quality of retrieved information; simple information augmentation is insufficient. Furthermore, the benefit of RAG is limited when a model’s intrinsic knowledge (eg, Gemma3:27B) already meets or exceeds the quality of the external knowledge base. This finding indicates that performance enhancement strategies must account for both the retrieval mechanism’s quality and the model’s preexisting capabilities.

## Introduction

### Background

With the rapid advancement of artificial intelligence (AI), medical chatbots are playing an increasingly pivotal role in providing personalized health care consultations and managing patient data [1,2]. However, despite the overall progress of large language models (LLMs), a performance gap between English and Korean persists, as technical breakthroughs in high-resource languages do not inherently transfer to midresource languages such as Korean [3,4]. These limitations act as structural constraints when directly applying general-purpose LLMs to the Korean health care environment, particularly in their failure to sufficiently reflect local medical systems, legal regulations, and ethical standards [5,6].

Concurrently, South Korea’s medical ethics, guidelines, and legal norms are closely integrated with highly standardized prescription and treatment protocols, which are intrinsically linked to the National Health Insurance reimbursement system, licensing requirements, and clinical guidelines from professional societies [7,8]. Consequently, standardized procedures and criteria often carry more weight than individual clinical discretion [9]. As a result, recommendations from general-purpose GPT-based models—primarily trained on United States or European guidelines—may conflict with Korean regulations, potentially suggesting interventions that are prohibited, not covered by insurance, or legally sensitive [10-12]. Such discrepancies pose significant risks, including information errors, misdiagnosis, and institutional misalignment, thereby compromising both patient safety and legal liability [13,14]. This misalignment represents a substantial barrier, revealing the structural limitations of applying general LLMs directly to the Korean health care context.

Furthermore, Korean medical inquiries are characterized by patients describing their symptoms, progress, and concerns in detailed narrative forms [15,16]. A primary challenge for medical chatbots is accurately extracting clinically essential information from such verbose text and matching it with the appropriate Korean medical guideline documents [17,18]. Specifically, as Korean is an agglutinative language with high morphological complexity—where medical vocabulary consists of a mixture of Hanja, loanwords, and native Korean terms—specialized text processing and retrieval technologies optimized for the Korean language are required [19,20].

Current LLM-based medical chatbots, including commercial models, primarily rely on pretrained internal knowledge. This often leads to “hallucinations”—generating factually incorrect information—and a failure to ensure reliability based on the latest domain-specific evidence [21,22]. Specifically, when consultation content pertains directly to a specific country’s insurance standards, local medical systems, or locally used products and ingredients, simple learning-based response mechanisms struggle to guarantee the precision and up-to-dateness required for medical safety [10]. To address these limitations, the ecosystem has recently shifted toward state-of-the-art open-source LLMs such as Llama 3 (Meta), Mistral (Mistral AI), and Gemma (Google LLC), which allow for higher levels of customization, including on-premises deployment, domain-specific fine-tuning, and enhanced data privacy [23]. Nevertheless, even high-performance open-source models require architectural reinforcement, such as systematic integration with external knowledge, to ensure accuracy and safety in the medical domain. In this context, retrieval-augmented generation (RAG) is emerging as a promising solution [24]. By using dense retrieval techniques and Korean-specific embedding models, RAG enables chatbots to access refined medical databases, allowing this study to focus on generating fact-based responses grounded in prevalidated medical literature, guidelines, and local regulations [25].

However, RAG alone faces limitations in sufficiently processing the unique characteristics of Korean medical inquiries. As these inquiries are often long and narrative, with the patient’s core intent frequently obscured by peripheral context (such as emotions or treatment experiences), simple vector similarity-based retrieval often fails to select the most appropriate evidence documents [26]. As a sophisticated enhancement to overcome this, metadata filtering has been proposed. This approach involves the LLM extracting structured metadata—such as clinical department, disease category, age group, and specific drugs or procedures—from the query and filtering the database accordingly before the retrieval stage, thereby narrowing the search space [27]. This ensures that the system performs retrieval and generation only on the most relevant subsets of documents, which significantly improves response accuracy and consistency, especially for complex, multistep medical questions [28]. Despite these developments, there is a lack of empirical research systematically comparing and evaluating the performance of various open-source LLM architectures that combine RAG with metadata filtering within the Korean medical context [29]. Furthermore, benchmarks and evaluation frameworks that reflect the unique characteristics of Korean medical inquiries are not yet sufficiently established [30]. We identified a research gap that necessitates an empirical validation of open-source LLM-based architectures integrating RAG and metadata filtering to design safe and reliable medical chatbots tailored to the Korean health care environment.

### Objectives

Therefore, we aimed to develop and comparatively evaluate a Korean medical consultation chatbot architecture that integrates RAG with LLM-based metadata filtering to better align responses with Korean clinical information needs and locally grounded evidence. We constructed a Korean medical document corpus (10.4 GB; 487,277 segments) and implemented an RAG pipeline using a Korean-specialized embedding model with an Elasticsearch-based retriever, augmented by metadata filtering that extracts structured clinical cues (eg, disease-, symptom-, or drug-related signals) to narrow the retrieval space before generation. We then compared 5 open-weight LLM backbones (Qwen3:4B, Mistral:7B, Llama-3.1:8B, Gpt-Oss:20B, and Gemma3:27B) under 3 configurations—baseline (no retrieval), RAG-only, and RAG plus metadata filtering—to determine when retrieval and filtering improve Korean health consultation responses. Performance was validated on 226 Korean consultation questions spanning 7 common disease categories, with an emergency medicine specialist rating each response for accuracy, safety, and helpfulness using a predefined scoring protocol.

## Methods

### Data Collection

This study focused on collecting and preprocessing Korean medical consultation data. To ensure a comprehensive dataset in diverse medical fields, we incorporated a variety of medical fields and consultation scenarios. The main data sources included publicly available disease information from the official websites of major Korean hospitals (n=3537; eg, disease encyclopedias accessible via the public health information portal of Asan Medical Center), legally usable open-source textbooks (n=2336), public data from the Ministry of Food and Drug Safety (n=87,635), and health-related knowledge data from major Korean search engines (n=49,681). These datasets were rigorously curated and refined through the supervision of health care professionals among the coauthors.

**Figure 1.**
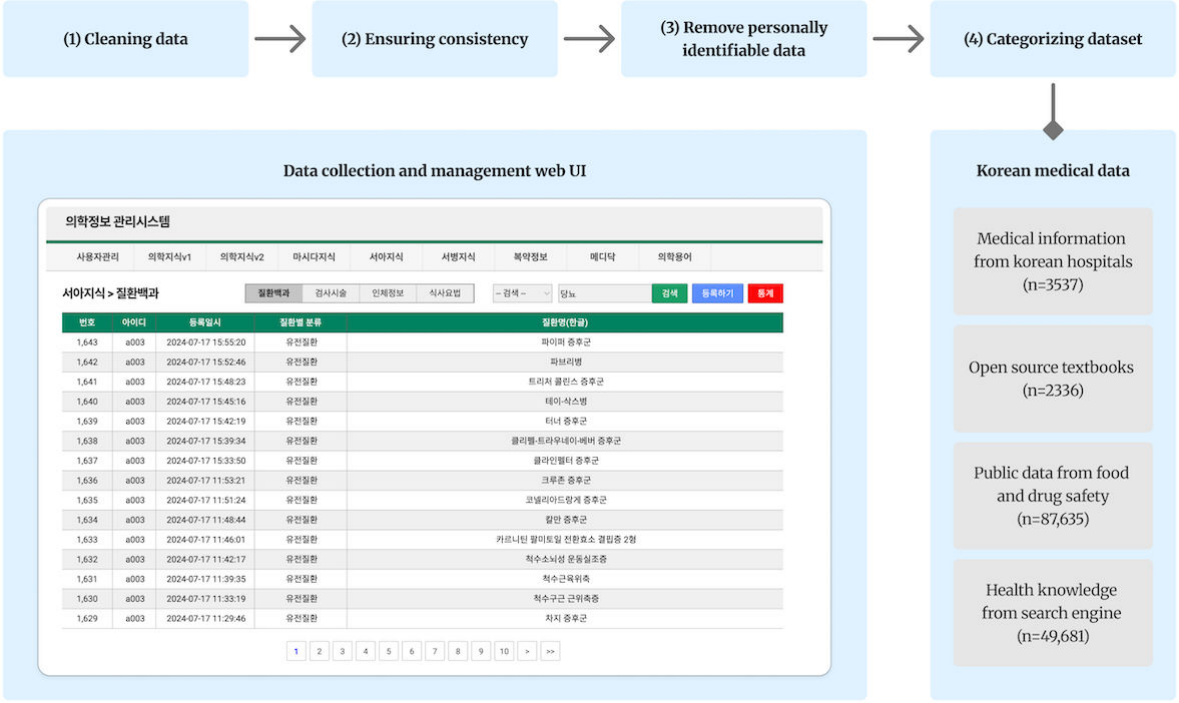
The database construction process, data count, and web UI screen for database management. We built a 10.4 GB medical document corpus, divided into 487,277 text segments, for efficient searching in the RAG system. RAG: retrieval-augmented generation; UI: user interface.

The data preprocessing process involved four key steps: (1) cleaning the data by removing unnecessary characters, duplicate entries, and nonmedical content to ensure dataset integrity; (2) ensuring consistency by correcting spelling errors, managing synonyms, and standardizing medical terminology; (3) conducting a rigorous screening process to ensure the complete absence of any personally identifiable information (PII) through a dual-verification approach, combining automated rule-based detection with manual review by medical professionals; and (4) classifying data by disease categories, examination and procedure types, body regions, medical specialties, and consultation types to enhance the efficiency of subsequent analyses. As a result, we constructed a medical document corpus of 10.4 GB, segmented into 487,277 text units for efficient retrieval within the RAG system. An overview of the data collection and management web user interface is shown in Figure 1.

This high-quality Korean medical consultation dataset, constructed through this process, is crucial for developing the RAG-integrated small large language models. This curated dataset includes frequently asked medical questions from the general public and answers based on the clinical expertise of medical professionals. By using this data for performance evaluation, we aimed to more accurately assess our service’s accuracy, safety, and helpfulness in a real-world medical consultation environment within Korea.

### Implementation of Default LLM

Concerning the LLM backbone, we built the LLM using Ollama as the backbone. The models were loaded from the Ollama library, allowing for model selection—including Qwen-4B (Alibaba Group), Mistral-7B (Mistral AI), Llama-3.1-8B (Meta), Gpt-Oss-20B (Google LLC), and Gemma-3-27B (Google LLC)—depending on the test. The service was run in an NVIDIA A100 (NVIDIA Corp) with 40 GB of video random access memory and an Ubuntu 22.04 (Canonical Ltd) environment to ensure the models could operate at optimal performance. Each model is briefly summarized in Table 1.

**Table 1.** Overview and characteristics of LLMs^a^ evaluated in this study. This table summarizes the LLMs^a^ compared in this research. The overall process is illustrated in Figure 2.

LLM	Parameter	Description	Selection reason
Qwen3:4B	4B	A multilingual LLM developed by China’s Alibaba Group that also offers respectable performance in the Korean language.	Chosen to validate the practical service applicability of a small large language model.
Mistral:7B	7B	An LLM developed by France’s Mistral AI, renowned for delivering high performance with a relatively small number of parameters.	A widely used small open-source model, often used as a performance benchmark for models in the 7B parameter class.
Llama-3.1:8B	8B	The latest generation open-source model from Meta. Widely recognized as a top-performing benchmark in the 8B class and is instruction-tuned.	Selected as a state-of-the-art benchmark for the 8B parameter class, given its widespread adoption and strong performance.
Gpt-Oss:20B	20B	OpenAI’s first open-source model to have its weights made public, known for its solid performance.	To evaluate the performance of the latest architectures and verify the on-device applicability of small-scale models at the 20B parameter level.
Gemma-3:27B	27B	Google’s latest generation instruction-tuned open-source model. Known for strong performance that competes with larger models.	To evaluate a recent, larger-scale model (27B) and compare its capabilities against the smaller models (4B-20B).

a LLM: large language model.

**Figure 2.**
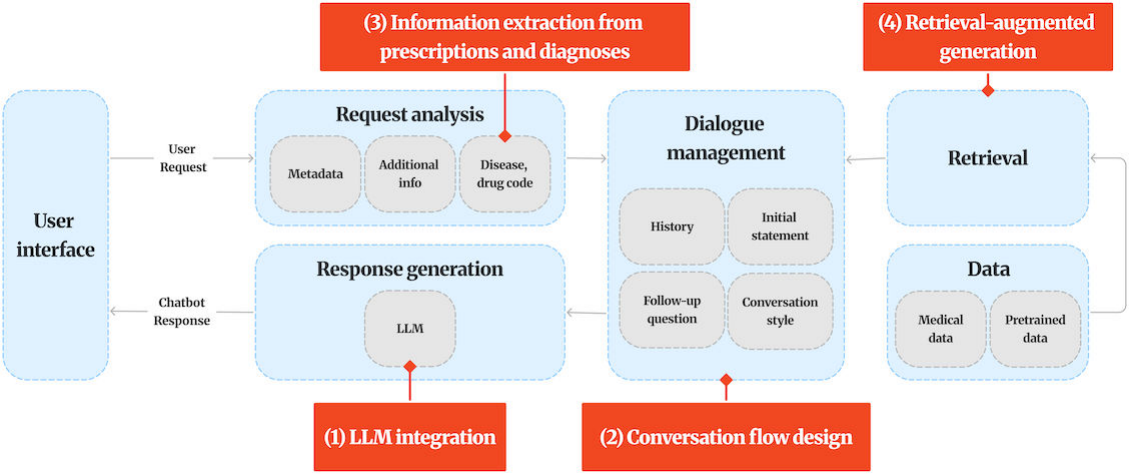
Implementation of a chatbot using a specific LLM. The diagram outlines the overall system architecture and workflow applied to develop the Korean health consultation chatbot. LLM: large language model.

Concerning conversation flow design, in health counseling, responding accurately and appropriately to user inquiries is crucial. Therefore, we carefully structured the conversation flow. This includes defining a step-by-step dialogue structure that incorporates initial greetings, symptom inquiries, requests for additional information, and guidance to visit a medical institution in case of an emergency, enabling users to interact smoothly with the chatbot. The chatbot also includes a memory function to remember the context of previous conversations and provide context-aware responses.

Concerning information extraction, when a user uploads an image of a prescription or medical report, the chatbot analyzes the image to extract medication and disease codes and presents the relevant information in a tabular format. This feature allows users to easily check detailed information about their health status and prescribed medications.

Concerning RAG, to use the database constructed in the data collection phase, we built an RAG and application programming interface system, enabling the LLM to generate answers based on this data. This approach ensures that the chatbot provides reliable and evidence-based information, consequently delivering more accurate and trustworthy medical responses to the user. A more detailed explanation of RAG will be provided in the following section.

### About RAG

Concerning Korean embedding, in this study, we adopted the upskyy/BGE-M3-Korean embedding model [31], which is specialized for the Korean language, to maximize the accuracy and efficiency of information retrieval. The key reason for selecting this model is that BGE-M3 possesses a “multigranularity” feature, making it specialized for Korean embeddings. This means it can process texts of various lengths—from short user queries to long, specialized medical documents—with consistently high performance. Furthermore, this model is fine-tuned on Korean data, enabling it to accurately capture complex Korean medical terminology and the subtle semantic nuances of user queries.

Concerning the Elasticsearch store configuration, for efficient information retrieval, we configured an Elasticsearch store, an open-source search engine designed to maximize search performance in large datasets using an inverted index structure [32]. In this study, it was integrated with a Korean medical database, allowing for fast responses to user queries. This setup enabled quick searches across large-scale medical data and maximized the performance of the RAG technique. Elasticsearch is particularly effective in text-based query-answering systems, offering fast and reliable search results.

Concerning retriever configuration, we constructed the retriever by integrating the aforementioned Korean embedding model with the Elasticsearch store. When a user inputs a query, the retriever uses the embedding model to generate a vector representation and searches the Elasticsearch store for the most similar vectors. These retrieved documents are provided as input to our backbone model, which then generates an optimal answer based on that data. Through this process, the system provides information based on reliable evidence rather than offering simple conversational answers. This ensures the chatbot can deliver accurate and trustworthy medical information to the user.

Concerning metadata filtering, we enhanced search efficiency using metadata filtering technology. Due to the nature of our model, which must use large volumes of health care data such as textbooks and accumulated big data responses, we determined that metadata filtering technology was essential for speed optimization. Metadata filtering first recognizes specific disease names or symptoms from the user’s query and uses relevant metadata to reconstruct the query more accurately. This improves search accuracy and helps users quickly access the information they want [33]. Table 2 shows examples of keywords that the metadata actually retrieves and the information brought in through them.

**Figure 3.**
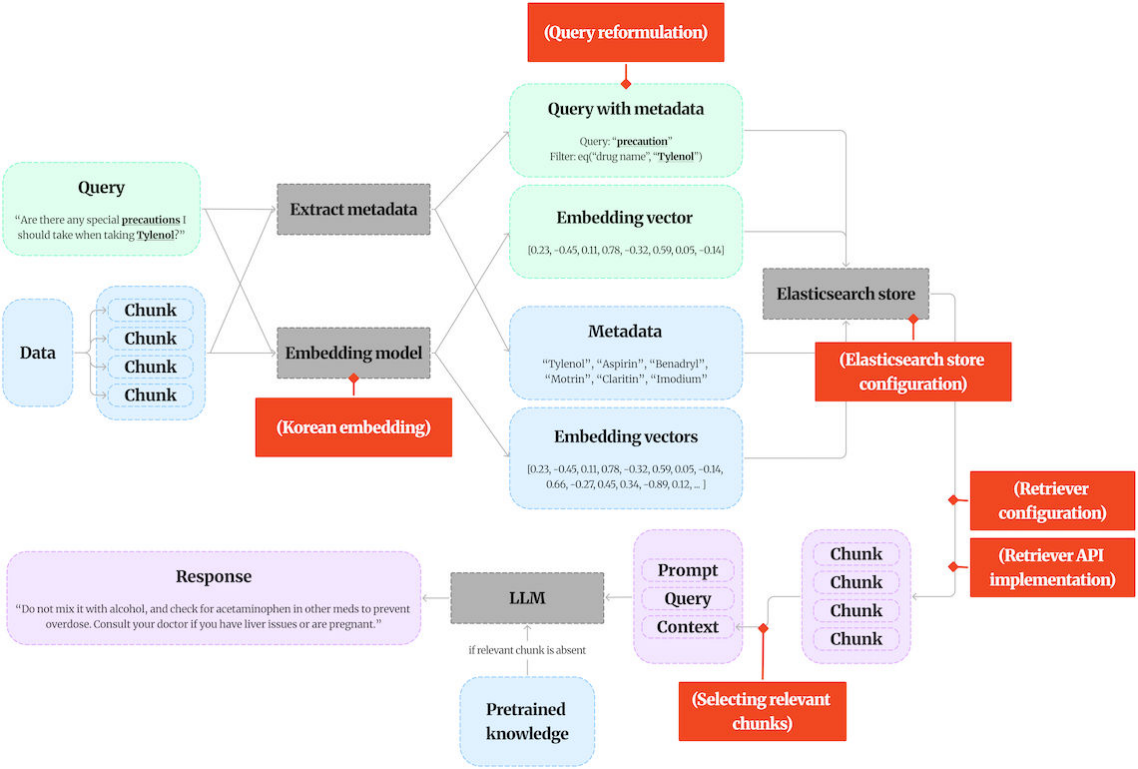
The RAG and metadata filtering diagram depicts the architecture of the RAG-based retrieval pipeline and API service, highlighting the integration of a Korean embedding model, Elasticsearch. API: application programming interface; LLM: large language model; RAG: retrieval-augmented generation.

Figure 3 depicts the overall workflow, illustrating the conversion of user queries into vector representations via the Korean embedding model for semantic retrieval.

**Table 2.** Comparison of data retrieved by the retriever with and without metadata filtering.

Data	Korean	English
Effect of metadata filtering: accurately includes “loss of motivation” and “depressive feelings,” which are the user’s core symptoms, and the explanation that this causes “psychosomatic symptoms.”		
Query	(중략) 치료는 되었는데 체중이 빠져 걱정되어 대장 내시경 검사를 했는데 결과는 용종 하나도 없이 깨끗한것으로 나왔습니다 문제는 체중감소와 컨디션 난조인데 건강에 상관 없는건지요? 약간의 우울증과 의욕이 좀 상실된 기분입니다 (중략)	(Abridged) treated... but worried about weight loss. Colonoscopy was clean... Problem is weight loss and poor condition... Also slight depression and loss of motivation... (abridged)
Extracted filter	체중 감소, 컨디션 난조, 우울증, 의욕 상실, 건강식품	Weight loss, poor condition, depression, loss of motivation, health supplements
Retriever without metadata filtering	[박리성 간질성 폐렴] (중략) 상 부위 자체도 폐포 대식세포들의 폐포 내 삼출이 주 소견이고, 간질은 거의 변화가 없거나 약하게 염증 세포 침윤만 관찰된다. 제2형 상피세포가 증가할 수도 있다. (중략)	[Desquamative interstitial pneumonia] (abridged) ...alveolar macrophages... interstitial changes are absent or mild... Type 2 epithelial cells may increase... (abridged)
Retriever with metadata filtering	[우울장애] (중략) 의욕 저하와 우울감을 주요 증상으로 하여 다양한 인지 및 정신 신체적 증상을 일으켜 일상 기능의 저하를 가져오는 질환 주의사항: 우울증, 즉 우울장애는 의욕 저하와 우울감을 주요 증상으로 하여 다양한 인지 및 정신 신체적 증상을 일으켜 일상 (중략)	[Depressive disorder] (abridged) a disease causing impairment in daily functioning with main symptoms of loss of motivation and depressive mood... Caution: Depression... causes various cognitive and psychosomatic symptoms... (abridged)
Effect of metadata filtering: “all-out sprint” or “strong labor intensity,” which are the user's key triggers, are directly linked to descriptions of physical overload symptoms such as “decreased athletic ability” and “difficulty breathing.”		
Query	(중략) 전력질주 2~30초.. 상하차같은 무거운걸 많이 나르는 노동강도가 강한 일을 할때 일시적으로 앞이 하나도 보이지 않게되고 귀가 먹먹해지는 증상이 나타나게 되었습니다.. 심지어 과음 후에 중간에 자다 깬적도 있는데 물마시러가다 앞이 안보이게되어 쓰러지듯 한적도 있습니다. (중략)	(Abridged) 20-30 seconds of all-out sprinting... heavy labor like loading/unloading... temporarily can't see anything and ears feel clogged... Even after heavy drinking... woke up... couldn't see while going for water and collapsed... (abridged)
Extracted filter	전력질주, 상하차, 노동강도, 앞이 안보이게, 귀가 먹먹해지다, 과음, 혈압 높다	All-out sprint, loading/unloading, labor intensity, can’t see, ears clogged, heavy drinking, high blood pressure
Retriever without metadata filtering	[유스타키오관] (중략) 엘리베이터가 상승하거나 하강할 때 귀가 먹먹해지는 것은 외부와 중이의 기압이 달라 고막이 비틀어지고 유스타키오관이 막히는 현상 때문입니다. 고막은 외부 환경과 신체 내부의 경계에 위치한 고막은 기압 차이에 의해 막이 터지는 것을 방지하기 위해 비틀어지면서 보호합니다. (중략)	[Eustachian tube] (abridged) ears feeling clogged when an elevator ascends or descends is due to the eustachian tube closing... the eardrum twists to protect itself from the pressure difference... (abridged)
Retriever with metadata filtering	[철결핍성 빈혈] (중략) 피로감, 무기력함, 운동능력 저하 등이 나타난다. 혈액이 산소 부족 상태가 되면 심장이나 폐가 이를 보충하기 위해 과도하게 활동해야 하므로 장기에 부담이 가해진다. 이에 따라 심장박동이 빨라지는 심계항진(palpitation; 두근거림)이나, 가슴의 통증, 호흡곤란 (중략)	[Iron-deficiency anemia] (abridged) fatigue, lethargy, decreased athletic ability, etc., appear. If the blood lacks oxygen, the heart or lungs must overwork... causing palpitation, chest pain, difficulty breathing... (abridged)
Effect of metadata filtering: directly matches ”diabetes,” the underlying condition of the mother provided by the user as key background information for the query.		
Query	어머니가 67세이신데 수액을 맞으셨는데 수액맞을때 카테타 니들에 피가 나왔는데 피가 심하게 검정색이였습니다. 어떤 증상이 있으면 그런지 알려주세요 어머니는 고혈압, 고지혈증, 당뇨가 있으십니다	Mother is 67... received an IV drip... blood came out of the catheter needle... the blood was severely black. Please tell me what symptoms cause this. Mother has high blood pressure, hyperlipidemia, and diabetes.
Extracted filter	고혈압, 고지혈증, 당뇨, 수액, 카테타, 피, 검정색	High blood pressure, hyperlipidemia, diabetes, IV drip, catheter, blood, black color
Retriever without metadata filtering	[유스타키오관] (중략) 엘리베이터가 상승하거나 하강할 때 귀가 먹먹해지는 것은 외부와 중이의 기압이 달라 고막이 비틀어지고 유스타키오관이 막히는 현상 때문입니다. 고막은 외부 환경과 신체 내부의 경계에 위치한 고막은 기압 차이에 의해 막이 터지는 것을 방지하기 위해 비틀어지면서 보호합니다. (중략)	[Seminal vesicle] (abridged) often discovered incidentally... can be caused by excessive proliferation of the seminal vesicle mucosa, inflammatory diseases like prostatitis... Hematospermia is treated symptomatically... (abridged)
Retriever with metadata filtering	[정낭] (중략) 본인도 잘 모르고 지내다가 우연찮게 발견되는 경우가 많은데, 정낭 점막의 과다한 증식, 전립선염 등의 염증성 질환에 의해 발생할 수 있습니다. 혈정액증은 증상에 따라 대증치료를 하며, 신체에 발생한 다른 원인질환을 치료하거나 정낭염의 경우 정낭을 마사지하고 항생제로 치료합니다. (중략)	[Noninsulin-dependent diabetes mellitus] (abridged) ...it is rare to be completely cured... However, diabetes can be managed... If diabetes is well-managed, a healthy life can be maintained... (abridged)

### Evaluation for AI Model Performance

To validate this chatbot system, we reconstructed 226 representative questions based on question and answer data provided by a medical consultation platform, focusing on 7 common diseases among Koreans: hypertension, acute bronchitis, diabetes, indigestion or gastritis, atopic dermatitis, allergic rhinitis, and reflux esophagitis. Table 3 summarizes the information about the question list.

The main question types by disease include lifestyle, medication, symptoms, and treatment. For scoring, an emergency medicine specialist provided the necessary answer guidelines for each question. We quantitatively assessed each model’s responses using 3 criteria: accuracy, safety, and helpfulness. We used the following prompt script to guide the assessment process (Textbox 1).

**Table 3.** Cross-tabulation of validation questions by disease category and question type. Statistics of the Q&A^a^ validation dataset for the verification of this chatbot system, consisting of 226 representative questions.

Disease or type	Lifestyle	Medication	Symptoms	Treatment	Complex	Total
Hypertension	8	9	11	7	0	35
Acute bronchitis	6	7	8	5	0	26
Diabetes	8	9	11	7	0	35
Indigestion or gastritis	6	7	8	5	0	26
Atopic dermatitis	3	7	8	5	0	23
Allergic rhinitis	6	7	8	5	0	25
Reflux esophagitis	7	7	8	5	0	26
etc	0	0	0	0	30	30
Total	42	53	62	39	30	226

a Q&A: question and answer.

Textbox 1. **Prompt script and criteria for the quantitative evaluation of artificial intelligence (AI) responses.**

Prompt script:

You are an expert evaluating the performance of a medical consultation AI system. Use the SCORING REFERENCE to assess the AI ANSWER’s content according to the following criteria:

Accuracy (1‐5 points)

Safety (1‐5 points)

Helpfulness (1‐5 points)

Please assign a score to the AI ANSWER for each criterion.

QUESTION: [Question statement]

SCORING REFERENCE: [Medically correct answer to serve as a reference for scoring]

AI ANSWER: [AI model’s response]

We established accuracy, safety, and helpfulness as the core criteria for evaluating AI responses. Accuracy comprehensively evaluates whether the AI’s answer is medically consistent with the model answer reviewed by a medical expert, and whether it clearly aligns with the user’s question intent. In parallel, safety assesses whether the answer contains dangerous information that could be harmful to the user, and whether it ensures the information’s reliability by including essential warning statements, such as “AI advice cannot replace professional medical consultation.” Helpfulness measures how easily the user can understand the accurate and safe information and receive practical help from it. In other words, an answer is considered good only when it goes beyond informational correctness and also possesses user-centered delivery and practicality.

### Ethical Considerations

All data used in this study were sourced exclusively from publicly accessible online platforms, including the official websites of major Korean hospitals, open-source medical textbooks, the Ministry of Food and Drug Safety, and Korean health search engines. These sources provide general disease-level medical knowledge intended for public health education and do not contain any patient-level records, clinical notes, or PII. No internal, proprietary, or private electronic health records were accessed.

As detailed in the Methods section, all collected data underwent a stringent verification process involving both automated screening and manual review by medical professionals to guarantee the total absence of any unintended PII. Accordingly, this study qualifies as research using publicly available information with no collection or recording of personal identifiers, and is exempt from Institutional Review Board review pursuant to Article 13, Paragraph 3 of the Enforcement Rule of the Bioethics and Safety Act in the Republic of Korea [34].

## Results

### Overview

The proposed model scored highest across accuracy, safety, and helpfulness. It significantly outperformed other models in terms of accuracy, safety, and helpfulness. The following table and graph visually represent the quantitative evaluation results for each model. To compare the performance differences between configurations (baseline vs RAG vs RAG+metadata filtering), we performed a paired *t* test for each model’s scores across the 226 validation questions.

Table 4 shows the results of analyzing the impact of RAG and metadata filtering technologies on the medical consultation response performance of various language models. The general trend indicates that applying RAG technology alone results in minimal performance improvement or even a slight decline. In contrast, when RAG and metadata filtering are applied together, most models show a significant performance enhancement.

**Table 4.** Analysis of quantitative evaluation results. This table presents the performance of each language model in terms of accuracy, safety, and helpfulness for medical consultation responses. *P* values were derived from 2-tailed paired *t* tests comparing each variant to its respective baseline model. Statistical significance was set at *P*<.05.

Models	Accuracy	Safety	Helpfulness	Average	*P* value
Qwen3:4B, mean (SD)	4.23 (0.06)	4.25 (0.05)	4.27 (0.05)	4.25 (0.04)	—^a^
Qwen3:4B+RA GB, mean (SD)	4.27 (0.06)	4.22 (0.05)	4.37 (0.05)	4.28 (0.04)	.40
Qwen3:4B+RAG^b^+metadata filtering, mean (SD)	4.41 (0.07)	4.25 (0.06)	4.47 (0.06)	4.37 (0.05)	.02
Mistral:7B, mean (SD)	3.51 (0.10)	3.74 (0.09)	4.14 (0.07)	3.79 (0.08)	—
Mistral:7B+RAG, mean (SD)	3.58 (0.13)	3.80 (0.12)	4.04 (0.10)	3.80 (0.11)	.73
Mistral:7B+RAG+metadata filtering, mean (SD)	3.92 (0.12)	4.13 (0.10)	4.25 (0.09)	4.10 (0.10)	<.001
Llama3.1:8B, mean (SD)	3.52 (0.08)	3.61 (0.09)	3.98 (0.06)	3.70 (0.07)	—
Llama3.1:8B+RAG, mean (SD)	3.50 (0.11)	3.58 (0.11)	3.85 (0.08)	3.64 (0.09)	.98
Llama3.1:8B+RAG+metadata filtering, mean (SD)	3.67 (0.10)	3.73 (0.11)	3.98 (0.07)	3.79 (0.09)	.04
Gpt-Oss:20B, mean (SD)	4.34 (0.04)	4.50 (0.04)	4.44 (0.06)	4.43 (0.05)	—
Gpt-Oss:20B+RAG, mean (SD)	4.41 (0.05)	4.57 (0.04)	4.39 (0.06)	4.46 (0.05)	.15
Gpt-Oss:20B+RAG+metadata filtering, mean (SD)	4.46 (0.04)	4.61 (0.03)	4.45 (0.06)	4.51 (0.04)	.03
Gemma3:27B, mean (SD)	4.38 (0.03)	4.32 (0.05)	4.56 (0.03)	4.42 (0.03)	—
Gemma3:27B+RAG, mean (SD)	4.31 (0.05)	4.26 (0.05)	4.43 (0.04)	4.34 (0.03)	≥.99
Gemma3:27B+RAG+metadata filtering, mean (SD)	4.49 (0.03)	4.34 (0.06)	4.61 (0.03)	4.48 (0.03)	.14

a Not available.

b RAG: retrieval-augmented generation.

The minimal effect of applying RAG alone appears to stem from the difficulty of accurately finding relevant and useful information from vast external data sources. Indeed, the change in average scores for the Qwen3:4B (4.25, SD 0.04 → 4.28, SD 0.04, *P*=.40) and Mistral:7B (3.79, SD 0.08 → 3.80, SD 0.11, *P*=.73) models was not statistically significant. Furthermore, the Llama3.1:8B and Gemma3:27B models actually experienced a decline in scores.

In contrast, when metadata filtering was combined, a clear improvement was observed, particularly in the midsized parameter models. The average score for the Mistral:7B model increased significantly from 3.79, SD 0.08, to 4.10, SD 0.10, and the Qwen3:4B model’s score rose from 4.25, SD 0.04, to 4.37, SD 0.05; both increases were statistically significant (*P*<.05). Gpt-Oss:20B also demonstrated a significant (*P*<.05) performance enhancement from 4.43, SD 0.05 to 4.51, SD 0.04, notably achieving the highest score of 4.61, SD 0.03 on the safety metric. This indicates that the effectiveness of RAG is highly dependent on the filtering process that controls the quality of the retrieved information.

**Figure 4.**
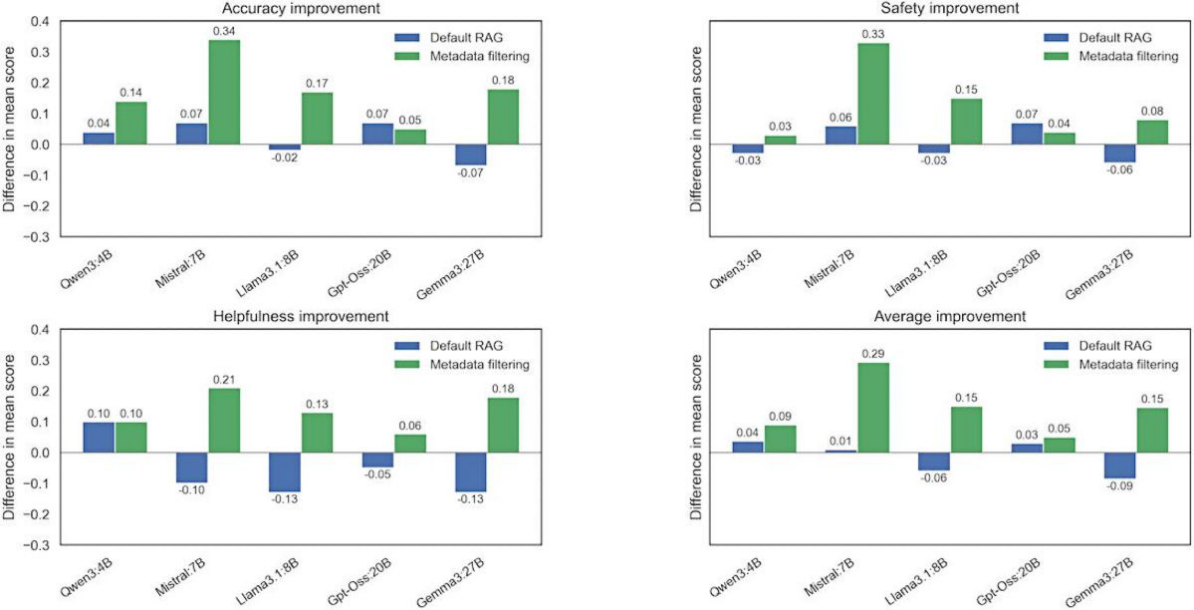
Graph of performance changes in the original model based on RAG and metadata filtering. RAG: retrieval-augmented generation.

Figure 4 compares the effects of RAG and metadata filtering against the original model. Each bar shows the average difference in accuracy, safety, and helpfulness compared to the baseline when RAG and metadata filtering are applied, with positive values indicating performance improvement. Overall, when the metadata filtering technique was included, the model showed improved performance in accuracy and helpfulness, which proves that it provides useful answers suited to the local Korean environment.

Statistical analyses were performed to evaluate the significance of performance changes. All continuous variables (accuracy, safety, and helpfulness) are reported as mean and SE of the mean. Differences between the baseline models and their RAG-augmented variants were assessed using a 2-tailed paired *t* test, assuming the scores for the same 226 questions are dependent. Statistical significance was defined as *P*<.05.

### Web Service Implementation

For this study, our development environment used Ubuntu 22.04 long-term support and an NVIDIA A100 40 GB graphics processing unit. We used the graphics processing unit for the computational operations of the LLMs. We applied Streamlit (Streamlit Inc) and LangChain (LangChain Inc) as development frameworks. Specifically, we used Streamlit to implement the web interface and LangChain to integrate and manage the LLMs.

**Figure 5.**
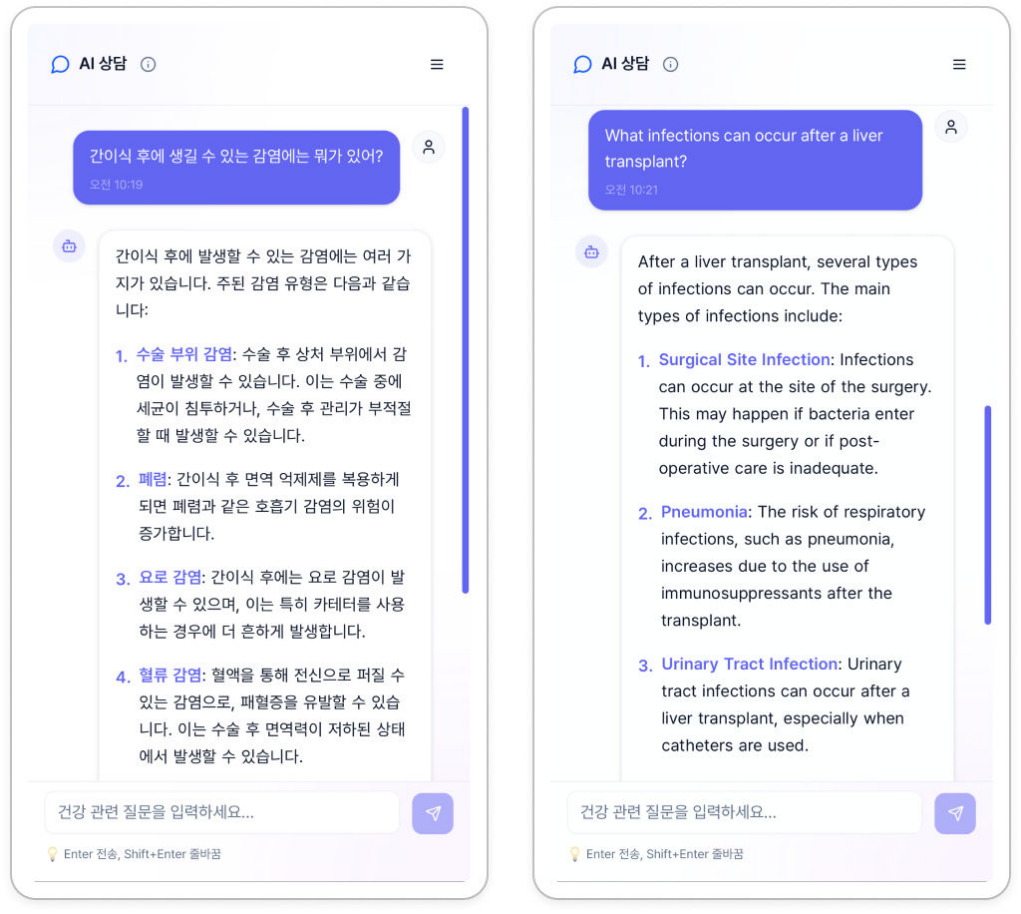
Screenshot of the implemented ”Geongangi” web service interface. The interface is designed to provide an intuitive user experience for both the general public and health care professionals, enabling easy access to health consultations.

The service developed through this process is named “Geongangi.” We aim for this service to provide information on disease symptoms, prevention, and health management, as well as posttreatment care conversations in the future. The intended target users are the general public and medical professionals. It can provide medical information to the general public and be used by medical professionals for the purpose of collecting patient information. Figure 5 shows the “Geongangi” web service interface, illustrating the user-friendly design that supports both patient and health care provider interactions.

## Discussion

### Principal Findings

This study aimed to determine under what conditions RAG and metadata filtering improve Korean health consultation responses across open-weight LLMs of varying scales. Our findings validate the hypothesis set in this study by revealing that retrieval augmentation yields meaningful gains only when retrieval quality is actively controlled through metadata filtering. Standalone RAG failed to produce statistically significant improvements in any model—and, in 2 cases (Llama3.1:8B and Gemma3:27B), resulted in score decreases—indicating that unfiltered retrieval over a large-scale Korean medical corpus introduces contextual noise that can degrade response quality. In contrast, RAG combined with metadata filtering produced statistically significant performance gains (*P*<.05) in 4 of the 5 models evaluated. Notably, the magnitude of improvement was inversely associated with model scale: smaller models such as Mistral:7B showed the greatest absolute gain (3.79, SD 0.08 → 4.10, SD 0.10), while the largest model, Gemma3:27B, showed no significant benefit (*P*=.14)—suggesting that beyond a certain knowledge threshold, external retrieval provides diminishing returns. Collectively, these results confirm that effective RAG deployment in specialized medical domains requires not merely an augmentation of information, but a deliberate mechanism for aligning retrieved content with the clinical structure of the target health care environment.

### Interpretations and Implications

The failure of standalone RAG to improve performance can be attributed to the unique linguistic characteristics of Korean medical inquiries. As demonstrated in Table 2, patient queries are frequently long and narrative, embedding core clinical signals within surrounding emotional expressions, personal histories, and contextual background. This structure creates a semantic mismatch between the patient’s actual clinical intent and the documents selected through vector similarity search alone; for example, a query describing weight loss, low mood, and loss of motivation was retrieved against a document on desquamative interstitial pneumonia rather than depressive disorder when filtering was absent. This finding is consistent with prior work identifying the limitations of dense retrieval in long, multitopic queries, where peripheral content dilutes the relevance signal and degrades retrieval precision.

Metadata filtering addresses this limitation not merely as a computational optimization, but as a mechanism that structurally aligns retrieval with the Korean clinical decision-making framework. South Korea’s health care system is characterized by highly standardized diagnostic and treatment protocols governed by National Health Insurance reimbursement criteria and professional society guidelines, in which institutional standards often take precedence over individual clinical discretion. As Korean medical knowledge is thus organized around well-defined categories in accordance with international standard guidelines—clinical department, disease classification, and drug and procedure type—ie, metadata filtering, which extracts these same clinical cues from the query before retrieval, naturally mirrors this organizational structure. This alignment explains why filtered RAG produced not only higher accuracy scores but also a meaningfully higher safety score in Gpt-Oss:20B (4.61, SD 0.03), whose reinforcement learning from human feedback–based alignment appeared to synergize with retrieved documents containing explicit clinical warnings and contraindication information.

Model-specific performance patterns further reveal that the benefit of metadata filtering is modulated by a model’s preexisting parametric knowledge. Mistral:7B, which showed the largest absolute improvement (3.79, SD 0.08 → 4.10, SD 0.10), appears to be well-suited to instruction-following but lacks the domain-specific knowledge to generate accurate Korean medical responses independently; metadata-filtered retrieval effectively compensated for this gap, representing the highest return on retrieval investment relative to model size. In contrast, Llama3.1:8B showed a score decrease under RAG-only conditions, likely because its limited context usage capacity caused unfiltered, noisy documents to interfere with generation rather than support it. Gemma3:27B, which exhibited no significant improvement even under filtered RAG (*P*=.14), likely reflects a knowledge ceiling effect—its 27B-scale pretraining corpus appears to have already internalized information equivalent to or exceeding the clinical scope of this study’s document database, rendering external retrieval marginally useful under current conditions. This effect may not generalize to domains requiring highly local, time-sensitive, or rare clinical knowledge not well-represented in general pretraining data.

These findings carry direct implications for practical deployment decisions. For resource-constrained settings that require on-premises deployment, smaller models (≤8B parameters) should be considered viable only in combination with high-quality metadata filtering, as their baseline medical knowledge is insufficient for safe clinical use. For midrange models (≈20B), the combination of filtered RAG and strong safety alignment offers a practical trade-off between computational cost and clinical reliability. For large models (≥27B), the marginal benefit of RAG may be limited unless the target domain involves highly specialized, locally specific, or frequently updated knowledge that is unlikely to be covered in pretraining data.

### Limitations

We identified several challenges and boundaries in this study that should be considered when interpreting the findings. First, the system continues to struggle with complex clinical inferences involving the simultaneous consideration of multiple medications or comorbid conditions, as the current metadata schema does not yet capture interaction-level clinical relationships. Second, the construction and maintenance of metadata tag sets requires substantial manual effort from domain experts, which may limit the system’s scalability when extending to rare or highly specialized medical subspecialties underrepresented in the current corpus. Third, real-world user query analysis revealed frequent inquiries regarding diet, exercise, and lifestyle management, indicating that future iterations must expand data coverage beyond structured clinical literature to include validated nutritional and behavioral health information. Finally, this study did not systematically analyze the downstream impact of metadata extraction errors—cases where the LLM incorrectly identified or omitted clinical cues from the query—on final response quality, and the error propagation characteristics of such failures remain an important area for future investigation.

### Conclusions

This quantitative evaluation confirms that architectural reinforcement through metadata filtering is essential for the reliable application of AI in the Korean medical domain. By demonstrating a framework that bridges the gap between general-purpose LLMs and the specific requirements of the local health care system, this study provides a blueprint for safer medical consultation tools. As these systems evolve to integrate broader health-related data, they will play a vital role in enhancing clinical efficiency and providing patients with high-quality, verified medical information that adheres to regional standards.

## Supplementary material

10.2196/72604 Checklist 1 Tripod+LLM checklist.

## Abbreviations

AI: artificial intelligence
LLM: large language model
PII: personally identifiable information
RAG: retrieval-augmented generation
